# Infertility Treatment and Umbilical Cord Length–Novel Markers of Childhood Epilepsy?

**DOI:** 10.1371/journal.pone.0055394

**Published:** 2013-02-13

**Authors:** Sari Räisänen, Arja Sokka, Leena Georgiadis, Maija Harju, Mika Gissler, Leea Keski-Nisula, Reetta Kälviäinen, Seppo Heinonen

**Affiliations:** 1 Department of Obstetrics and Gynaecology, Kuopio University Hospital, Kuopio, Finland; 2 Department of Pediatric Neurology, Kuopio University Hospital, Kuopio, Finland; 3 University of Eastern Finland, Kuopio, Finland; 4 Information Department, Welfare and Health Data Resources Unit, National Institute for Health and Welfare, Helsinki, Finland; 5 Nordic School of Public Health, Gothenburg, Sweden; 6 Department of Neurology, Kuopio University Hospital, Kuopio, Finland; University of Montreal, Canada

## Abstract

**Background:**

Epilepsy is one of the most common neurologic disorders of childhood, affecting about 0.4−0.8% of all children up to the age of 20.

**Methodology:**

A population-based retrospective cohort study. Aim was to determine incidence and identify perinatal and reproductive risk factors of epilepsy in children born between 1989 and 2008 among women (n = 43,389) delivered in Kuopio University Hospital. Risk factors of childhood epilepsy were determined by using logistic regression analysis.

**Principal Findings:**

The incidence of childhood epilepsy was 0.7% (*n* = 302 of 43,389). Maternal epilepsy, major congenital anomalies and use of assisted reproductive technology (ART) were associated with 4.25-, 3.61-, and 1.67- fold increased incidence of childhood epilepsy. A 10 cm increase in umbilical cord length was associated with a 15% decrease in the incidence of epilepsy (adjusted OR 0.85, 95% CI 0.78−0.94). However, the above reproductive factors accounted for less than 2% of total incidence, whereas maternal epilepsy proved to be the highest risk factor.

**Conclusions:**

Perinatal and reproductive factors were shown to be minor risk factors of childhood epilepsy, implying that little can be done in obstetric care to prevent childhood epilepsy. Infertility treatment and umbilical cord length, independent of gestational age and congenital malformations, may be novel markers of childhood epilepsy.

## Introduction

Epilepsy is one of the most common neurologic disorders of childhood, affecting about 0.4−0.8% of all children up to the age of 20 [1]–[3]. The incidence of childhood epilepsy is highest in the first year of life [1]. The etiology of epilepsy is poorly understood, but central nervous system (CNS) infections and anomalies [1], [4], major non-CNS anomalies [1], [4], metabolic conditions [4] and head trauma [1], [3] have been shown to be risk factors. Abnormal development of the brain during prenatal life may be a causal factor in some of these neurological disorders. Accordingly, previous studies have identified several perinatal factors associated with increased incidence of epilepsy. The risk profile of epilepsy includes maternal preeclampsia [5], [6], eclampsia [4], [6], maternal infections during pregnancy [4], [7], intrauterine fetal growth restriction [8], being small for gestational age [4], having a low Apgar score at birth [9], [10], and being either preterm [8] or post-term at birth [8], [11]. The risk of developing epilepsy is 4−10% in close relatives, especially siblings and offspring [1], implying that genetic factors are clear risk factors for childhood epilepsy. On the other hand, pregnancy and neonatal outcomes of women with active epilepsy have been shown to be equivalent to the general population when a predefined protocol was used for follow-up in antenatal and neurologic care [12]. To date, there have been only a limited number of population-based studies into events during pregnancy and delivery and their possible association with the subsequent incidence of childhood epilepsy among offspring. Thus, we reviewed retrospective population-based register data to identify perinatal and reproductive risk factors of childhood epilepsy among the total population of women with singleton live births in Kuopio University Hospital between 1989 and 2008.

## Methods

### Objectives

We analyzed data gathered from the Kuopio University Hospital Birth Register for the period 1989 to 2008. The data included information on maternal and neonatal birth characteristics and perinatal outcomes (live- and still-born infants born after the 22nd week of pregnancy or weighing 500 g or more). In addition, information on background, such as previous surgeries, illnesses, obstetric history, contraceptive use, smoking habits and alcohol consumption was collected using a self-administered written questionnaire. The information was complemented by midwife interviews conducted during visits or at delivery in the Kuopio University Hospital.

Congenital anomalies were unspecified and included only major congenital anomalies, including structural abnormalities. Children with medication treated childhood epilepsy were determined based on the register of the Social Insurance Institution of Finland, which was linked to Kuopio University Birth Register using children’s unique identification numbers. The Social Insurance Institution (SII) of Finland maintains a national registry of reimbursed medicines purchased by Finnish citizens. The anti-epileptic drugs (AEDs) are fully reimbursed, and qualification for this refund is based on clinical diagnosis (ICD-10 codes available since 2000 onwards) and a statement provided on a semi-structured form by a pediatric or adult neurologist. The right to receive fully refundable standard AEDs is granted by the SII to non-institutionalized patients. Children with epilepsy who are permanently institutionalized receive drugs free-of-charge from the institution. Placentas and umbilical cords (weight, length and insertion of the umbilical cord) were screened clinically by midwives using identical procedures after every delivery, either vaginal or Cesarean. Placental abruption and placenta previa were diagnosed by clinical examination or ultrasonography as previously described [13], [14]. Estimation of gestational age was based on data for the last menstrual period, unless there was a discrepancy of more than seven or 14 days at the first- or second-trimester ultrasonography measurements, respectively.

Birthweight was considered small for gestational age (SGA) when the sex and gestational age adjusted birth weight was below the normal tenth percentile [15]. Low birthweight (LBW) was defined as a birth weight less than 2500 g. Apgar scores were considered low if they were 0−6 at 1 or 5 minutes. Umbilical cord length was expressed as a standard deviation (SD) from the gestational-age and sex-specific mean values according to our own growth curve measurements. Assisted reproductive technology (ART) included in-vitro fertilization (IVF), intracellular sperm injection (ICSI), frozen embryo replacement and egg donation, but the rationale for selection of a particular ART method was not detailed in the obstetric database used. Women who had been hospitalized for observation because of an episode of bleeding after the 20^th^ gestational week were classified as having late pregnancy bleeding. Body mass index (BMI) was calculated by dividing body weight in kilograms by the squared height in meters (kg/m^2^). Placental/fetal mass ratio was calculated as placental weight in grams divided by the weight of newborn in grams×100. Preeclampsia was defined as gestational hypertension (≥140/90 mmHg) with proteinuria (≥500 mg in a 24-hour period) after the 20^th^ gestational week. Gestational diabetes mellitus was defined by high blood glucose levels according to oral 75-g glucose tolerance tests (fasting≥4.8 mmol/l, after 1 hour ≥10.0 mmol/l, and after 2 hours ≥8.6 mmol/l) during pregnancy. Elevated liver enzymes were defined as previously described [16]. Anemia was defined as hemoglobin levels below 120 g/L (WHO). Amnionitis was based on the ICD-10 code set clinically by obstetricians at the time of birth. Cigarette smoking and alcohol consumption during pregnancy and infertility problems were self-reported variables.

### Participants

The data corresponded to the total population (n = 43,389) of singleton live births during the study period from 1989 to 2008 taking place in the Kuopio University Hospital, which is the tertiary level perinatal center of the area. Multiple pregnancies (n = 1,768) and stillborn fetuses (n = 193) were excluded from the analysis. It is known that multiple pregnancies carry higher risk for complications and we wanted to avoid the bias towards possible higher risk towards epilepsy caused by these pregnancies. On the other hand, there were too few multiple pregnancies to be analyzed separately.

### Ethics

The Kuopio University Hospital gave its permission to use its patient register data to create the research database. The Social Insurance Institution of Finland granted permission to use their data in this study and to link the data on epilepsy to the study register. The Data Protection “Ombudsman” gave his statement before the study data was created, as requested by the national legislation on data protection. All childbearing women gave written informed consent for the collection of study data and its subsequent use in scientific research at the time of data collection. The study was approved by the Ethics Committee of Kuopio University Hospital.

### Statistical Methods

Differences between the study group (302 children with epilepsy) and the reference population (*n* = 43,087) were assessed by using Chi-square and Mann-Whitney U tests as appropriate. Differences were deemed to be significant if *p*<0.05. 95% confidence intervals (CI) were also calculated. The incidence of epilepsy was assessed by logistic regression analyses using a forward elimination procedure. Possible statistically significant confounding factors (p*<0.05*) were identified from maternal, fetal and delivery characteristics and delivery interventions. Furthermore, to examine whether background characteristics (maternal epilepsy, maternal age, parity, ART or umbilical cord length), major congenital anomalies, low Apgar score (≤6) at birth or mode of delivery are related to childhood epilepsy, the contribution of each of these factors was estimated using logistic regression. Each confounder was added separately to model B and the contribution of each factor was measured by the percentage reduction in the odds ratio of childhood epilepsy. The formula used was: (OR Model B – OR Model X)/(OR Model B –1) [17].The data were analyzed using SPSS for Windows 19.0 Chicago, IL.

## Results

The incidence of registered epilepsy among the 43,389 children born following singleton pregnancies in the Kuopio University Hospital between 1989 and 2008 was 0.7% (*n* = 302). The incidence of epilepsy was highest in the first year of life, i.e., 14.2% (*n* = 43 of 302), while the mean age (±SD) was 5.5 (±4.5) years. Significantly more children with epilepsy were conceived by ART (1.1% vs. 0.7%, *p* = 0.02) and born by Caesarean section with lower Apgar scores, as shown by the results of the univariate analyses in Tables 1 and 2. Children with epilepsy also had a higher risk of being born as SGA and with LBWs as well as a greater incidence of major congenital anomalies (3.0% vs. 0.8%, *p*≤0.001). They also tended to have on average a lower birthweight (mean±SD; 3381.8±782.1 g vs. 3508.7±600.0 g, *p* = 0.07) compared to children without epilepsy.

**Table 1:** Newborn characteristics among children with and without epilepsy.

Characteristic	With epilepsy	Without epilepsy	*p* value
	0.7% (*n* = 302)	(*n* = 43,087)	
Male	52.6	51.1	0.58
Female	47.4	48.9	
Mean birthweight±SD (g)	3381.8±782.1	3508.7±600.0	0.07
≤2499	10.6	4.5	≤0.001
2500–2999	10.6	10.3	
3000–3499	29.1	31.3	
3500–3999	29.8	35.6	
≥4000	19.9	18.4	
SGA (<90 percentile)	13.9	10.1	0.03
Mean birth height±SD (cm)	49.8±3.1	50.0±1.8	0.68
Mean head circumference±SD (cm)	34.8±2.5	35.0±1.8	0.19
Apgar score at 1 min. ≤6	12.9	4.9	≤0.001
Apgar score at 5 min. ≤6	4.6	2.0	0.001
Venous pH ≤7.15	5.2	3.2	0.14
Arterial pH ≤7.15	18.1	15.6	0.39
Major congenital anomaly	3.0	0.8	≤0.001

SGA = small for gestational age, SD = standard deviation.

**Table 2:** Pregnancy and delivery characteristics in women with and without a child with epilepsy.

Characteristic	With epilepsy	Without epilepsy	*p* value
	0.7% (*n* = 302)	(*n* = 43,087)	
Mean maternal age±SD (year)	29.6±5.7	29.0±5.5	0.04
Mean number of previous pregnancies	1.6±1.9	1.5±1.6	0.13
Mean number of previous deliveries	1.2±1.5	1.0±1.3	0.14
Primiparous women	35.8	40.9	0.07
Multiparous women	64.2	59.1	
Pregravid BMI±SD	23.2±4.3	23.3±4.7	0.65
Mean weight increase±SD (kg)	12.9±5.1	13.5±5.3	0.05
Mode of delivery			
Vaginal	67.2	77.2	≤0.001
Vacuum	4.6	6.0	
CS, total	28.2	16.8	
CS, elective	11.6	7.7	
CS, non-elective	16.6	9.1	
Mean gestational age at birth±SD (weeks)	38.8±3.1	39.3±2.1	0.19
≤27	2.0	0.5	≤0.001
28−31	2.7	0.7	
32−36	5.8	4.7	
37−40	67.8	69.8	
41−42	21.0	23.9	
≥43	0.7	0.3	
Epidural analgesia in vaginal deliveries	26.2	36.7	0.002
Placental abruption	2.0	0.6	0.003
Preeclampsia	4.3	3.8	0.63
Elevated liver enzymes	0.0	0.8	0.12
ART	1.1	0.7	0.02
Of which IVF	0.7	0.7	0.91
Maternal bleeding during pregnancy	2.4	1.6	0.35
Amnionitis	1.7	1.1	0.31
Maternal epilepsy	3.3	0.8	≤0.001
Maternal smoking			
Before pregnancy	16.3	24.6	0.18
During pregnancy	12.2	11.9	0.94
Maternal anemia			
First trimester	0.3	0.2	0.59
Second trimester	0.7	0.8	0.75
Third trimester	1.3	1.0	0.53
Maternal hypertension	2.6	2.0	0.39
Umbilical cord insertion			
Central	83.3	80.4	0.24
Marginal	14.0	17.5	
Velamentous	2.7	2.1	
Mean umbilical cord length±SD (cm)	55.7±12.0	58.7±13.0	≤0.001
Lowest 5%, ≤38	7.9	4.1	0.001
39−43	6.3	5.8	
44−74	79.5	78.4	
75−81	4.6	6.6	
Longest 5%, ≥82	1.7	5.2	

BMI = body mass index, CS = Cesarean section, ART = assisted reproductive technology.

**Figure 1:**
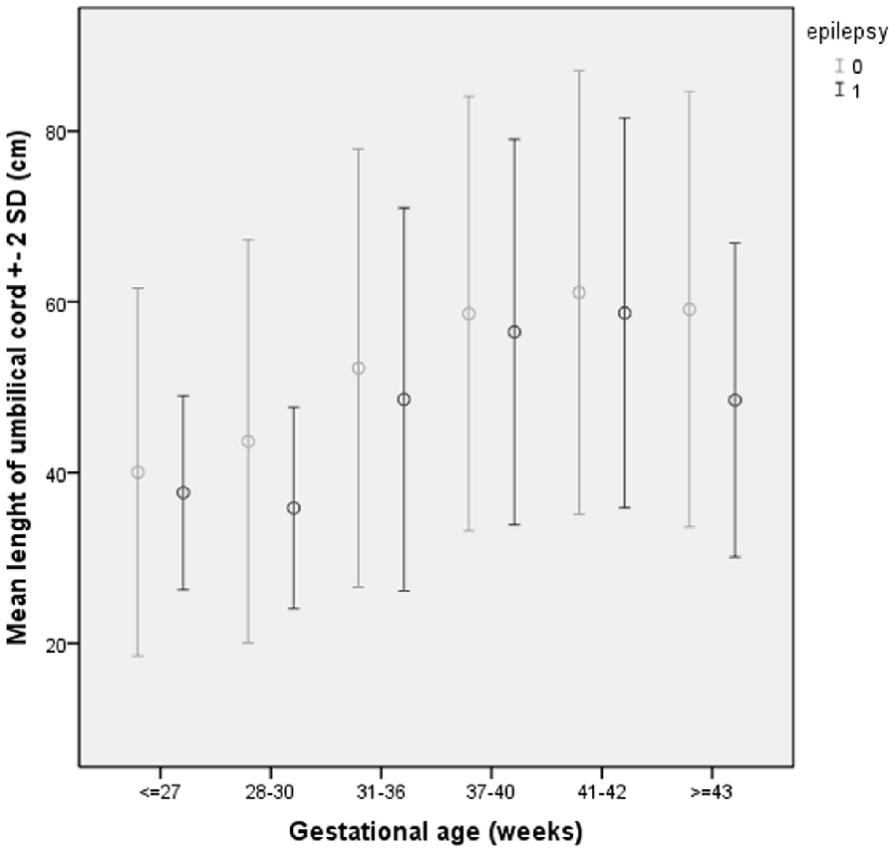
Mean umbilical cord length (±SD) in women with and without a child with epilepsy at different gestational age.

Maternal characteristics and reproductive risk factors were similar for the study group and the reference population except for age, parity, mode of delivery, maternal epilepsy, gestational age and use of epidural analgesia (Table 2). Out of the total population of women, 0.8% (*n* = 356) suffered from epilepsy at the time of pregnancy, and the incidence of childhood epilepsy among their offspring was significantly higher compared to unaffected women (2.8% vs. 0.7%). Significantly more children with epilepsy were born after gestations with abrupted placenta and with short umbilical cords. The mean length of the umbilical cord±SD was 3 cm shorter (55.7±12.0 cm) in the study group than in the reference population (58.7±13.0 cm), *p*≤0.001. There were significant differences in umbilical cord length among women with and without epilepsy at different gestational stages (*p*≤0.001) (see Figure 1.) Further, the mean SD of the umbilical cord length relative to gestational age among these groups varied significantly (−0.20 vs. −0.01, *p* = 0.002).

After adjustment for the selected confounding factors, maternal epilepsy, use of ART in index pregnancy, and major congenital structural abnormalities were independently associated with a 4.3-, 1.7-, and 3.6-fold higher incidence of epilepsy among offspring, respectively, compared to children without such characteristics (Table 3). Further, among children born by elective or non-elective-CS incidence of childhood was 1.5- and 1.9-fold higher than among children had spontaneous vaginal delivery. Interestingly, the incidence of epilepsy decreased by 15% (adjusted OR 0.85, 95% CI 0.78−0.94) per 10 cm increase in the length of umbilical cord.

**Table 3:** Adjusted ORs of childhood epilepsy (*n* = 42,322).

Delivery intervention (%)/characteristic	aOR (95% CI)	*p* value
Vaginal	1	
Vacuum	0.84 (0.48–1.48)	0.55
CS, elective	1.52 (1.05–2.20)	0.03
CS, non-elective	1.86 (1.35–2.56)	≤0.001
Major congenital anomalies	3.61 (1.83–7.10)	≤0.001
Umbilical cord length (10 cm)	0.85 (0.78–0.94)	0.001
ART	1.67 (1.05–2.64)	0.03
Maternal epilepsy	4.25 (2.24–8.08)	≤0.001

OR adjusted for maternal epilepsy, mode of delivery, ART, maternal age (continuous), gestational age (categorical), birthweight (continuous), Apgar score at 5 minutes, malformation, placental abruption and cord length (continuous).

CS = Cesarean section, ART = assisted reproductive technology.

In addition, we measured the contribution of maternal epilepsy, mode of delivery, Apgar scores at five minutes, ART, congenital anomalies and umbilical cord length to the incidence of epilepsy based on the percentage reduction in the odds ratio, as shown in Table 4. The odds ratio of epilepsy was 4.23 (95% CI 2.23−8.02) after adjustment for maternal epilepsy alone, and it increased to 4.34 (95% CI 2.29−8.24) after adjustment for parity and maternal age. Further, after umbilical cord length was added to model B, the odds ratio decreased to 4.30 (95% CI 2.26−8.15). This means that only 1.2% of the epilepsy risk can be explained by umbilical cord length, and overall the contribution of the above-mentioned factors ranged from 0 to 2.4%.

**Table 4:** ORs of childhood epilepsy after adjustments for characteristics and risk factors.

	Model A	Model B	Model C		Model D		Model E		Model F		Model G	
				Diff. with B (%)*		Diff. with B (%)*		Diff. with B (%)*		Diff. with B (%)*		Diff. with B (%)*
Childhood epilepsy	4.23	4.34	4.26	2.4	4.30	1.2	4.34	0	4.29	1.5	4.30	1.2
95% CI	(2.23–8.02)	(2.29–8.24)	(2.25–8.09)		(2.27–8.15)		(2.29–8.24)		(2.26–8.13)		(2.26–8.15)	

* (The contribution of each factor was measured by the percentage reduction in the odds ratio of epilepsy compared to Model B by using formula (OR Model B – OR Model C/D/E/F/G)/(OR Model B –1).

Model A = Adjusted for maternal epilepsy.

Model B = Adjusted for Model A+maternal age and parity.

Model C = Adjusted for Model B+mode of delivery.

Model D = Adjusted for Model B+Apgar score ≤6.

Model E = Adjusted for Model B+ART.

Model F = Adjusted for Model B+Major congenital anomalies.

Model G = Adjusted for Model B+umbilical cord length.

## Discussion

The incidence of registered epilepsy among children (*n* = 43,389) born after singleton pregnancies in the Kuopio University Hospital during 1989−2008 was 0.7% (*n* = 302). The incidence of epilepsy was 4.3-fold higher in children whose mothers were affected by epilepsy. Use of ART and major fetal abnormalities were associated with a 1.7- and 3.6-fold increased risk of childhood epilepsy, respectively. The mean length of umbilical cords appeared to be 3 cm shorter in children with epilepsy than in the reference population. The association between umbilical cord length and incidence of epilepsy was confirmed by the multivariate analysis, which revealed that the incidence of epilepsy decreased by 15% (adjusted OR 0.85, 95% CI 0.78−0.94, *p* = 0.001) per 10 cm increase in the length of umbilical cord.

The overall incidence of epilepsy in childhood was 0.7%, which was in close agreement with the expected rates in Finland reported earlier [18], [19]. We found that there was an increased incidence of childhood epilepsy associated with maternal epilepsy, congenital anomalies and use of ART of 4.3-, 3.6- and 1.7-fold, respectively. Our results concerning genetic risk and anomalies are in line with previous results [1], [4]. The results of a population-based study in Canada suggested that major non-CNS anomalies and CNS anomalies are associated with a 2.2- and 5.7-fold increased risk of epilepsy, respectively [4]. However, our data did not include specifics of the major structural abnormalities, and therefore we were not able to study the influence of congenital anomalies in more detail. Further, the observed association between ART and increased incidence of childhood epilepsy is supported by previous register-based studies from Sweden and Denmark [20], [21]. These studies suggested that the incidence of epilepsy was 1.50-fold higher (95% CI 1.10–2.15) in infants born after IVF and 1.71-fold higher (95% CI 1.21–2.42) if the woman had received infertility treatment. It might be argued that these results could be explained by reduced fertility among women with epilepsy [22], and thus a greater dependence on ART. However, in the present study, there was no difference in the frequency of use of ART between women with and without epilepsy (0.9% vs. 0.8%, *p* = 0.85, data not shown), which agrees with the reported lack of difference in fertility between women with and without epilepsy in the same population for the period 1989 to 2000 [24].

We found that the mean length of umbilical cord was significantly shorter in children affected by childhood epilepsy compared to the reference population. It has previously been shown that the length of the umbilical cord was associated with fetal sex and socioeconomic status [25]. It has also been shown that umbilical cord length is a measure of the fetal moves and tensile forces [26] and thus, with increasing parity women give birth to newborns with longer umbilical cords due to larger uterine cavity [27]. A short umbilical cord has been shown to be a potential marker of intrauterine events that can place the neonate at a risk of future adverse developmental events [25], [28] such as mental and motor impairments [25], congenital anomalies, urinary tract abnormalities [29] and death that was shown to be two-fold among infants with congenital anomalies [30].

Our results may be biased by multicollinearity since among children affected by childhood epilepsy there were significantly more congenital anomalies than among the reference population (3.0% vs. 0.8%, p≤0.001, respectively) and furthermore congenital anomalies are known to be a risk factor of a short umbilical cord. However, after adjustment for anomalies, the length of umbilical cord was an independent risk factor even though based on the present study the pathogenesis remains unclear and challenges new studies. Epilepsies are a heterogeneous and etiologically multifactorial group of brain diseases. In future, we aim to continue the work to identify the exact nature of the anomalies associated with the increased risk of epilepsy. It is also important to identify the types and etiologies of epilepsies associated with maternal epilepsy, the use of ART as well as short umbilical cord length.

Our findings regarding the lack of a link between maternal preeclampsia or eclampsia and childhood epilepsy are contrary to the results of previous studies, which suggested such complications are associated with an increased risk of epilepsy [4]–[6]. This might be explained by differences in treatment practices and study populations since eclampsia is very rare in Finland but was diagnosed in 0.3% of the cases considered in the USA and Canada.

Overall, our results showed that perinatal and reproductive factors accounted for only about 1−2% of the total incidence of childhood epilepsy, indicating that most perinatal complications play a minor role in the development of childhood epilepsy. In contrast, genetic factors, such as maternal epilepsy, were found to pose the greatest risk, and thus it is likely that very little can be done in obstetric care to prevent childhood epilepsy.

### Strengths and Weaknesses

The most important strength of the present study was that the data covered the entire population of children delivered between 1989 and 2008 in the Kuopio University Hospital. In Finland, over 99% of women give birth in public hospitals. Several clinical studies have been conducted to validate the data gathered from the Kuopio University Hospital Birth Register [13], [14], [31]–[33]. Epilepsy diagnoses registered by the SII of Finland for the purposes of drug reimbursement are verified by a pediatric or adult neurologist, and thus are likely to be reliable. The Finnish SII registry does not cover the small minority of people (3% in 2003, data on the SII file) with epilepsy who, although eligible, elect not to use the fully refundable drug system. The registry also does not cover patients who permanently reside in an institution. Further, a small minority of patients in the registry could have been misdiagnosed as having epilepsy. However, it has recently been estimated that the registry covers 97% patients of all patients with newly diagnosed epilepsy [18].
